# High Prevalence of *Helicobacter pylori hopQ* II Genotype Isolated from Iranian Patients with Gastroduodenal Disorders

**DOI:** 10.1155/2014/842469

**Published:** 2014-02-10

**Authors:** Amin Talebi Bezmin Abadi, Ashraf Mohabbati Mobarez

**Affiliations:** ^1^Department of Medical Microbiology, Utrecht University, Heidelberglaan 100, 3584 CX Utrecht, The Netherlands; ^2^Department of Bacteriology, Faculty of Medical Science, Tarbiat Modares University, Tehran, Iran

## Abstract

*Helicobacter pylori* plays an important role in the pathogenesis of chronic gastritis, peptic ulceration, and noncardia gastric cancer. Several putative virulence factors for *H. pylori* have been identified including *vacA*, *babA*, and *iceA*. HopQ is one of the outer membrane proteins involved in bacterial adherence to gastric mucosa and has been suggested to also play a role in the virulence of *H. pylori*. Due to the substantial geographic differences in the prevalence of *H. pylori* virulence factors reported, the main purpose of the current study was to investigate the association between different *H. pylori* virulence *hopQ* alleles (types I and II) and patients with gastroduodenal disorders. The presence of *H. pylori* and *hopQ* alleles in gastric biopsy specimens was identified by specific PCR assays. *H. pylori* type II *hopQ* was found to be significantly associated with gastric cancer patients (odds ratio: 3.47, 95% CI: 1.56–5.89). Information about the prevalence of *H. pylori hopQ* type II can be used for determining the high-risk diseases type which is actually colonized by *H. pylori hopQ* type II positive strains. The presence of *H. pylori hopQ* type II should be investigated in different geographical regions as confirmatory findings may provide a definite biomarker attributed to the pathogenesis of certain severe digestive diseases.

## 1. Introduction

*Helicobacter pylori* is a gram negative and successful gastric pathogen which colonizes more than 50% of the world population [1]. Infection with *H. pylori* usually happens in the first few years of life, persisting for life if untreated [2]. *H. pylori* infection is the main cause of gastric and duodenal ulcers, as well as a potential risk factor for gastric cancer and mucosa-associated tissue lymphoma [3]. It has been proposed that only in a certain number of individuals that *H. pylori* infection will progress to severe gastroduodenal diseases [4]. Due to frequently occurring point mutations, *H. pylori* infections are heterogeneous [5]. *H. pylori* strains are thought to possess various virulence factors which contribute to the digestive disease sequelae [6–8], yet so far they remain ill-defined [9, 10]. To date, available reports indicate a slight association between gastroduodenal diseases and *H. pylori* virulence factors [11]. Over the past decade, while different determinants have been reported as virulence factors influencing the progression to different diseases [12–14], studies are often contradictory [6, 15–18].

Rapid advances in sequencing technology and whole genome analysis enable researchers to search for novel virulence factors. In 2005, Cao et al. reported that *H. pylori hopQ* genotypes are associated with an increased risk for peptic ulcer disease [19]. HopQ is an outer membrane protein involved in bacterial adherence to the gastric mucosa [20]. Loh et al. showed that, in certain *H. pylori*, adherence to the gastric epithelial cells are slightly facilitated in strains expressing HopQ [21], though they did not provide further data about disease specific virulence factor of *hopQ*. The high rate of *H. pylori* infection in Northern Iran and the increasing number of digestive complaints lead to the current study on whether the presence of *hopQ* (type I or II) can affect disease outcome. The main aim of this study was to investigate the possible association between two major *H. pylori hopQ* alleles by studying clinical samples isolated from patients with various gastroduodenal diseases using PCR.

## 2. Materials and Methods

### 2.1. Patients

Included in this study were patients undergoing upper gastric endoscopy due to different digestive orders, from which *H. pylori* clinical isolates were obtained. The study was approved by the Ethics Committee of Tarbiat Modares University, Tehran, Iran. Written informed consent was obtained from all patients prior to entering the study. Participants were divided into four patient groups: duodenal ulcer, gastric ulcer, gastritis, and gastric cancer. Inclusion criteria for patients with documented *H. pylori* infection were (i) positivity in *H. pylori *culture, (ii) not having reported antibiotics or omeprazole consumption in the previous 5 months, and (iii) being at least 18 years old. Endoscopic observations and histology findings were used to determine the diagnosis for each patient who had *H. pylori* strains isolated [22]. Last biopsy specimen was kept frozen at −70°C until further analysis.

### 2.2. *H. pylori* Strains

Two antral biopsies were inoculated in sterile thioglycolate broth (Merck, Germany) and shipped in cold temperature (5°C) to the diagnostic laboratory for routine bacterial culture according to the standard method. Culture medium was Colombia agar supplemented with 7% sheep blood, amphotericin B (5 mg/mL), trimethoprim lactate (5 mg/L), vancomycin (10 mg/mL), and polymixin-B (2500 units/mL) and incubated in microaerophilic conditions of 10%CO_2_, 85%N_2_, and 5%O_2_ at 37°C for 10 days with 95% humidity [23]. Organisms were identified as *H. pylori *by Gram staining, colony morphology and positive oxidase, catalase, and urease reactions. All *H. pylori* single colony strains were stored in −80°C in Brucella broth (Merck, Germany) supplemented with 25% (FCS) (Gibco, CA, USA) and 20% glycerol.* H. pylori* ATCC 43504 was used as a reference strain in this study.

### 2.3. PCR Analysis

Genomic DNA was only extracted if a single colony was available, or as a result of positive culture and urease tests. DNA extraction was performed using the genomic DNA QIAamp DNA mini kit (Qiagen GmbH, Hilden, Germany) according to the manufacturer's instructions. The presence of *H. pylori* DNA was confirmed by PCR targeting the phosphoglucosamine mutase gene, *glmM* [24]. In this experiment, separate PCRs were performed for *hopQ *type I and *hopQ* type II using the 187 and 160 bp primer sets, respectively (Table 1) [25]. 6 *μ*L of each PCR product was loaded onto 2% agarose gels (Sina-Gene, Tehran, Iran), stained with ethidium bromide. *H. pylori *strains 26695 and J99 were included as positive control strains.

### 2.4. Statistical Analysis

IBM SPSS Statistics version 21.0 was used for statistical analysis. A *P* value of <0.05 was considered as statistically significant.

## 3. Results

Genomic DNA from 300 antrum biopsy specimens during 2007–2009 was purified successfully. The distribution of clinical diagnoses was as follows: 75 (25%) with gastritis, 48 (16%) with gastric cancer, 87 (29%) with gastric ulcer, and 90 (30%) with duodenal ulcer. The mean age was 39 years (range 21–73 years) and the percentage of females was 41%.

No significant associations were observed between age and gender or clinical manifestations of all four investigated patient groups (*P* > 0.05). By PCR, *hopQ *type I was present in 100 of 300 (33%) strains and *hopQ *type II was found in 156 of 300 (52%) strains. Furthermore, 44 (15%) *H. pylori* isolates were found to be negative for *hopQI *and *hopQII*, so they were excluded from analysis. *H. pylori* type* II hopQ *was significantly found to be predominant in patients with gastric cancer (odds ratio: 3.47, 95% CI: 1.56–5.89) (Table 2). In contrast, no significant association was observed between disease type and *hopQ *type I.

## 4. Discussion

Clinical development of *H. pylori* infection is affected by the interaction of several virulence factors as well as by the host. *H. pylori* infection is the main causative agent of superficial gastritis and shows an inevitable role in the etiology of peptic ulcer disease [26]. In regard to biologic concepts, achieving successful and long term colonization requires complex adhesion mechanisms for bacteria. Hence, all possible bacterial products were under focus for investigating the likely contribution in bacterial colonization. *H. pylori hopQ* is one of the main outer membrane proteins on the bacterial surface and is the biggest outer membrane protein family observed in *H. pylori* genome. Determining a link between *H. pylori hopQ* and certain digestive diseases may provide a start point for answering questions regarding *H. pylori* adherence to gastric cells. This study was designed to determine the frequency of *H. pylori hopQ* genotypes isolated from biopsy specimens. Our findings demonstrate a high prevalence of *H. pylori hopQ* type II genotype among Iranian patients with gastric cancer. To our knowledge, this is the first report on the prevalence of *H. pylori hopQ* alleles, in infections among symptomatic Iranian individuals. It has been suggested that specific genotyping-based analysis of *H. pylori* isolates can be useful for predicting postinfection disorders [3, 27]. Recent studies have shown that the *hopQ* type I allele is strongly associated with an increased risk of peptic ulcer diseases (PUD) in western countries [28] and that *H. pylori hopQ II* is frequently detected among investigated population [28]. Furthermore, outer membrane proteins of *H. pylori* have shown a strong potential for increasing the severity of related gastroduodenal disorders. Ohno et al. [28] did not identify any relationship between *hopQ* type I and II alleles and other virulence factors such as *cagA* and *vacA* in terms of clinical outcomes. However, the exact association between virulence factors of *H. pylori* and *hopQ* alleles needs further investigation especially in genetically different populations. In an investigation by Ohno et al., [28] the prevalence of *hopQ *I among gastritis and gastric cancer patients reported 58% and 68%, respectively. However, our results indicate that the frequency of *hopQ* I was similar in both duodenal ulcer and gastritis patients (36% and 29%, resp.). Our findings have shown that *hopQ* I is the less prevalent genotype among the *H. pylori* isolates recovered from the Iranian population (Table 1). In contrast to a study from United States [20] which reported a significant association between the carriage of *H. pylori hopQ* type I among the peptic ulcer patients, Ohno et al. [28] did not identify a relationship between both *hopQ* alleles and clinical outcomes of infection (*P* > 0.05). It has been reported that *hopQ* type II strains have low frequency (less than 1%) among the Far East countries. Data from western countries indicate that the prevalence of *hopQ* type II strains (36%) is more common than countries from eastern Asia (1%). In conclusion, this study showed that *hopQ *II genotype is frequently present in *H. pylori* strains isolated from gastric cancer patients in Iran. It was the first report regarding the clinical relevance of *H. pylori hopQ *II, a basis on which further studies should be conducted. If further studies confirm our findings, this could lead to improved diagnostic strategies for clinicians to fight the current dogma for gastric cancer in those countries.

## Figures and Tables

**Table 1:** Primer sequences, PCR condition.

Amplified allele	Primer sequences	Thermal condition	Cycles	Reference
Type I *hopQ *	5-CAACGATAATGGCACAAACT-3 5-GTCGTATCAATAACAGAAGTTG-3	94°C for 60 seconds, 54°C for 45 seconds, and 65 seconds for 72°C	35	[25]

Type II* hopQ *	5-TCCAATCCAGAAGCGATTAA-3 5-GTTTTAATGGTTACTTCCACC-3	94°C for 60 seconds, 54°C for 45 seconds, and 65 seconds for 72°C	35	[25]

**Table 2:** Distribution of the *hopQ *I and *hopQ *II among the different disease.

Disease groups	*hopQ *I			*hopQ* II		
	Positives	Odd ratio	95% CI	Positives	Odd ratio	95% CI
Gastritis (*n* = 75) (control group)	22 (29%)	Reference		30 (40%)	Reference	
Gastric ulcer (*n* = 87)	40 (45%)	0.61	0.50–1.03	42 (48%)	0.81	0.91–1.21
Duodenal ulcer (*n* = 90)	32 (35%)	0.79	0.17–2.43	39 (43%)	0.5	0.21–1.79
Gastric cancer (*n* = 48)	6 (12%)	0.54	0.09–2.23	45 (93%)	3.47	1.56–5.89
